# Challenging the fixed-criterion model of perceptual decision-making

**DOI:** 10.1093/nc/niad010

**Published:** 2023-04-20

**Authors:** Jennifer Laura Lee, Rachel Denison, Wei Ji Ma

**Affiliations:** Center for Neural Science and Department of Psychology, New York University, 4 Washington Pl, New York City, NY 10003, United States; Center for Neural Science and Department of Psychology, New York University, 4 Washington Pl, New York City, NY 10003, United States; Department of Psychological & Brain Sciences, Boston University, 64 Cummington Mall, Boston, MA 02139, United States; Center for Neural Science and Department of Psychology, New York University, 4 Washington Pl, New York City, NY 10003, United States

**Keywords:** subjective inflation, perceptual decision-making, criterion-setting, signal detection theory

## Abstract

Perceptual decision-making is often conceptualized as the process of comparing an internal decision variable to a categorical boundary or criterion. How the mind sets such a criterion has been studied from at least two perspectives. One idea is that the criterion is a fixed quantity. In work on subjective phenomenology, the notion of a fixed criterion has been proposed to explain a phenomenon called “subjective inflation”—a form of metacognitive mismatch in which observers overestimate the quality of their sensory representation in the periphery or at unattended locations. A contrasting view emerging from studies of perceptual decision-making is that the criterion adjusts to the level sensory uncertainty and is thus sensitive to variations in attention. Here, we mathematically demonstrate that previous empirical findings supporting subjective inflation are consistent with either a fixed or a flexible decision criterion. We further lay out specific task properties that are necessary to make inferences about the flexibility of the criterion: (i) a clear mapping from decision variable space to stimulus feature space and (ii) an incentive for observers to adjust their decision criterion as uncertainty changes. Recent work satisfying these requirements has demonstrated that decision criteria flexibly adjust according to uncertainty. We conclude that the fixed-criterion model of subjective inflation is poorly tenable.

## Introduction

We must often make judgments about what we see, from categorizing a person in the distance as a friend or stranger to deciding whether a faint sense of motion in our periphery was really something moving or in fact nothing at all. Such perceptual decisions require that we produce a categorical answer based on the available visual information. Perceptual decision-making is often conceptualized, therefore, as the process of comparing an internal decision variable to a categorical boundary or criterion.

The question of how human observers set such a criterion has received two apparently conflicting answers, each with broader theoretical implications. On the one hand, studies have proposed that people are only able to maintain one criterion (Gorea and Sagi 2000, 2001, Rahnev et al. 2011, 2012, Ko and Lau 2012, Li et al. 2018)—sometimes called the “unique criterion” (Gorea and Sagi 2001) or “common criterion” (Morales et al. 2015) model. The studies in this group have tended to use weak (near-threshold) stimuli, and their findings have been linked to consciousness-related phenomena like subjective inflation, where it has been proposed that only one criterion is used at a given time (Rahnev et al. 2011, Solovey et al. 2015, Rahnev et al. 2012, Odegaard et al. 2018, Knotts et al. 2019, Abid 2019, Lau and Brown 2019) and blindsight, where it has been proposed that the criterion is fixed across time (such that it never adjusts to the weaker visual signals following visual cortical damage) (Ko and Lau 2012). In consciousness science, the nature of the criterion has important theoretical consequences. Threshold crossing—the process of surpassing a criterion—has been associated with certain kinds of mechanisms for the generation of conscious perception, like “ignition” (Fisch et al. 2009, Noy et al. 2015) and higher-order decisional or metacognitive processes that attribute consciousness to sensory signals of sufficient strength (Kang et al. 2017, Pereira et al. 2022, Ko and Lau 2012, Denison et al. 2022). For example, proponents of a “higher-order thought” (HOT) theory of consciousness propose that a higher-order representation is needed to make a first-order perceptual state conscious (Lau and Rosenthal 2011). Importantly, the model favored by some higher-order theorists assumes that the criterion is fixed. For example, Lau and Brown write, “Because human subjects can only use the same criterion for both the attended and unattended if they are presented simultaneously (a known psychophysical fact based on previous work Gorea and Sagi (2000)), the higher variability of the internal signal under the lack of attention turns out to lead to more frequent crossing of the criterion, i.e. more frequent occurrence of subjective perception.” (Lau and Brown (2019), Blockheads!, p. 180) Evidence for this view has come from the finding that visual phenomenology is often inflated above what would be predicted based on sparse sensory content (Odegaard et al. 2018): observers report peripheral (Solovey et al. 2015) or unattended (Rahnev et al. 2011) stimuli as “seen” more often than would be expected based on the observers’ performance in discriminating features of those stimuli. For threshold-crossing views of consciousness, the notion of a single, fixed criterion is intuitive and appealing.

Other studies, however, have proposed that people can flexibly adjust their criteria, applying different decision rules to categorize different stimuli depending on the context and the observer’s goals (e.g. Qamar et al. 2013, Denison et al. 2018, Whiteley and Sahani 2008, Maiworm et al. 2011, Green and Swets 1974). These studies have often used suprathreshold stimuli with a task of deciding whether a stimulus belongs to one category or another, and the findings have indicated strategic adjustment of criteria depending on prior information about the more likely category, the relative rewards of the two categories, and sensory uncertainty. For example, studies have demonstrated that observers incorporate uncertainty information when integrating prior knowledge with uncertain sensory information (Acerbi et al. 2014, Körding and Wolpert 2004, Jazayeri and Shadlen 2010). The cue combination literature provides further evidence that uncertainty information is represented and used in perceptual decision-making: many studies demonstrate that observers integrate noisy cues from multiple sensory modalities in a near-optimal way, where each cue is weighed in accordance with its reciprocal variance (Alais and Burr 2004, Körding et al. 2007, Van Beers et al. 1999, Knill and Saunders 2003). Moreover, subjects incorporate uncertainty information in many tasks requiring higher-level visual cognition (Zhou et al. 2020, Shen and Ma 2016, Qamar et al. 2013, Ma et al. 2011, Yang et al. 2016). For normative views of decision-making, the idea of flexible, context-dependent criteria is intuitive and appealing.

Only a few studies have designed experiments intended to test whether criteria are fixed or flexible, and conclusions have been mixed. Foundational work by Gorea *et al*. found that false alarm rates for detecting a threshold contrast stimulus (Gorea and Sagi 2000) or contrast increment (Gorea and Sagi 2001) differed when a stimulus was presented together with another stimulus of different contrast vs. alone. To account for these data, Gorea *et al*. proposed that in multi-stimulus displays, observers could not maintain separate internal response distributions or criteria for decision-making, leading to a fixed absolute criterion.

Subsequent studies of near-threshold perception adopted the unified criterion model and found that it was qualitatively consistent with their findings. An influential study by Rahnev et al. (2011) manipulated attention to different simultaneously presented stimuli and found more conservative signal detection criteria for attended vs. unattended stimuli when d’ was matched across attention conditions. That is, although performance was equated, observers were more willing to report that a stimulus was present when it was unattended than when it was attended. This finding was called “subjective inflation” because observers felt like they saw more than what was explainable by their performance. Later work found further evidence for subjective inflation in detection tasks (Odegaard et al. 2018, Solovey et al. 2015, Li et al. 2018). Subjective inflation, in turn, became a key piece of motivating data for higher order theories of consciousness, due to the dissociation between objective and subjective performance (Lau and Brown 2019, Brown et al. 2019). The original Rahnev *et al*. paper proposed an elegant model of subjective inflation based on the unified criterion idea of Gorea *et al*.: a fixed absolute criterion could explain subjective inflation. Subsequent studies of subjective inflation adopted this model (Morales et al. 2015, Solovey et al. 2015, Odegaard et al. 2018). As a result, the idea of a fixed criterion for conscious perception gained a foothold.

The unified criterion conclusion arrived at by (Gorea and Sagi 2001) was later criticized, however, on the grounds that the noise distribution in different contrast conditions could not be known by the experimenter and may not have been constant, as Gorea *et al*. assumed (Kontsevich et al. 2002). When the noise variance is unknown, inferring the absolute criterion from false alarm rates is not possible, so the question of whether the criterion is fixed or flexible cannot be resolved (see also Denison et al. (2018)). A few subsequent studies worked to address this issue of model unidentifiability using external noise to place the noise distributions under experimental control. These studies generally found suboptimal but flexible criteria. In these studies, uncertainty has been manipulated using contrast (Qamar et al. 2013, Adler and Ma 2018), luminance (Zak et al. 2012), eccentricity (Zhou et al. 2020), and orientation variability (Rahnev et al. 2011). Some studies have found evidence for “criterion attraction”, where criteria from different uncertainty conditions are closer to each other than would be optimal, although not identical (Zak et al. 2012, Rahnev et al. 2021). Meanwhile, an external noise study that manipulated uncertainty via inattention found evidence for flexible and near-optimal criteria under different attentional states (Denison et al. 2018). In summary, when experimental methods are designed to distinguish fixed from flexible criteria, the evidence has tended to favor flexible criteria.

This still leaves open a puzzle, though, as to whether the body of research, largely focused on consciousness, that has proposed or incorporated the idea of a fixed criterion can be reconciled with work that demonstrates flexible (though often suboptimal) criteria. To address this issue, we have selected two very similar studies to use as case studies for comparison. The first is the original Rahnev *et al*. subjective inflation study, which proposed the fixed criterion model of subjective inflation. The second is Denison *et al*., which also manipulated attention but used external noise to measure absolute criteria and found evidence for flexible criteria. We have chosen to focus on Rahnev et al. (2011) and Denison et al. (2018) because both investigated spatial attention. Rahnev et al. (2011) was the original study to provide evidence for subjective inflation under inattention and to explain it with a fixed criterion. As the concept of subjective inflation has been particularly influential to the consciousness science community, we have highlighted that paper here. These studies have potentially important differences—notably, Rahnev *et al*. used a detection task with near-threshold stimuli whereas Denison *et al*. used a categorization task with suprathreshold stimuli. But here we sought to determine whether their seemingly opposite conclusions could be reconciled by methodological and analytical considerations alone.

To do so, we mathematically identified the space of parameter combinations consistent with behavioural evidence used to support the “fixed criterion” hypothesis in Rahnev *et al*. 2011 and show that this space in fact contains a large set of “flexible criterion” solutions. Here, we show that the empirical evidence presented by these studies is consistent with a broader class of generalized Bayesian observers that take uncertainty into account when setting decision criteria. We conclude that previous proposals that subjective inflation arises from a fixed decision rule are therefore not strictly supported; both sets of findings are consistent with a flexible decision criterion that accounts for attention. Finally, we describe how the methods used by Denison *et al*., which go beyond the standard signal detection theoretic framework, allowed inferences about decision rules that were not possible in previous studies—lending strength to the evidence for flexible criterion-setting.

## Background

### Signal detection theory

**Figure 2.**
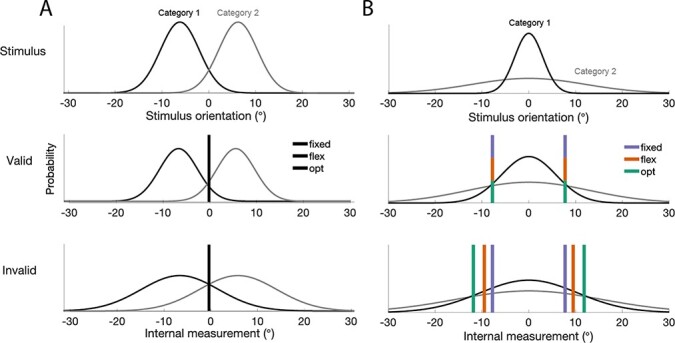
(A): Standard signal detection theoretic task. For discrimination or categorization tasks in which the category distributions are symmetric and offset (e.g. where category 1 produces an internal decision variable distribution with mean $-\mu$ and variance *σ* and where category 2 produces a distribution of mean *µ* and equal variance *σ*), there is typically no incentive for the optimal observer to shift their decision criterion as trial-to-trial variability increases. The optimal decision criterion continues to bisect the means of the two category distributions, thus failing to provide an optimal observer with an incentive to shift their decision criterion as uncertainty changes. (B): The embedded category task. In a variant of the standard fine-discrimination task, observers categorize an oriented stimulus drawn from one of two orientation distributions: a narrow distribution (category 1) or a broad distribution (category 2), both centered around horizontal (0 degrees), such that when the orientation of the stimulus is near horizontal, it is more likely to be from category 1. As variance increases from the valid to invalid condition, decision criteria may stay fixed (purple), adjust flexibly (e.g. square root relation, orange), or adjust optimally (green).

In signal detection theory (SDT), a visual stimulus is internally mapped to a one-dimensional decision variable in the observer’s head, corresponding—in theory—to whichever dimension of the stimulus which is task-relevant. In a stimulus detection paradigm, the subject is instructed to respond about whether they believe the stimulus was “present” or “absent” on a given trial. Repeated presentations of the same stimulus (e.g. a Gabor with the same contrast strength) are thought to result in a decision variable with some amount of trial-to-trial variability (*σ*) (Green and Swets 1974). A trial with a low decision variable value should be classified as “unseen,” and one with a high value should be classified as “seen.” Each internal decision variable distribution for “present” or “absent” stimuli is a theoretical distribution formed from infinite repeated observations of stimulus-present or “stimulus-absent” trials. These two decision variable distributions overlap one another, resulting in some degree of inherent ambiguity for any one given observation. The decision-making system resolves this by setting a decision boundary (also called a decision criterion): an observation should be classified as “absent” unless it yields an decision variable value greater than a given decision criterion, in which case it should be classified as “present”. If the decision-making system has perfect metacognitive access to the true mean (*µ*) and variance (*σ*) of the two decision variable distributions, then it should be able to compute the optimal decision criterion—that is, the threshold at the intersection of these two distributions that maximizes decision accuracy over many trials (Fig. 2, panel A).

SDT is a very general framework, and the decision variable can range from a basic sensory signal to a highly derived cognitive quantity. The decision variable is often referred to, abstractly, as the “strength of evidence” for one decision category or another. Green and Swets (Green and Swets 1974) conceptualized the decision variable as a log posterior ratio, a comparison of the probabilities of each category. To actually calculate such probabilities, one needs a generative model describing how stimulus inputs are transformed into internal responses. In perception science, it is often possible to specify such a generative model, in which a stimulus generates a noisy internal “measurement”, which can then be used to determine the probability of each stimulus given that internal response. This measurement is “absolute” in the sense that it is considered to be a physical quantity in the brain that is directly related to the stimulus (e.g. neural firing rate or estimated orientation). The fixed-criterion proposal refers to the measurement space: the idea is that on each trial observers make a decision by comparing their sensory measurement to some fixed value.

The first step in formalizing a signal detection theoretic model is to define the statistics of the observations. Part of the generative model is defined by the task at hand: the stimulus *s* takes two discrete values—on a detection task, the stimulus is either present or absent. If the stimulus is present, *s* has a fixed, experimenter-set value, which we will denote by *µ*. If it is absent, we will define it as 0. The two stimulus values are equally likely on any given trial, so that


(1) $$ p(s=0) = p(s=\mu) = 0.5. $$

To complete the generative model, we have to specify the nature of the observations. We assume that on each trial, the observer makes a noisy measurement *x* of the stimulus *s*. As is standard in signal detection theory (and motivated by the central limit theorem) we assume the noise to be zero-mean Gaussian noise. Thus, we have:


(2) $$ p(x|s) = \frac{1}{\sqrt{2\pi\sigma^2}} e^{-\frac{(x-s)^2}{2\sigma^2}}. $$

If the signal (stimulus) is absent, then the decision variable is drawn from a normal distribution with mean 0 and standard deviation *σ*. If the signal is present, then the variable is drawn from a normal distribution with mean *µ* and standard deviation *σ*. The distributions *p*(*s*) and $p(x|s)$ fully define the generative model.

Let us say that on a given trial, the measurement of the stimulus is *x*_trial_ and the observer is asked to infer whether the stimulus is present, i.e. to infer *s*. Given the generative model, the log posterior ratio over *s* is then


(3) $$\begin{aligned} \text{LPR} & = \log \frac{p(x_{\text{trial}}|s=\mu)}{p(x_{\text{trial}}|s=0)} \end{aligned}$$

(4) $$\begin{aligned} & = \frac{\mu}{\sigma^2}\left(x_{\text{trial}}- \frac{\mu}{2}\right), \end{aligned}$$

which is a multiple of the measurement itself. Therefore, it is, under this generative model, equivalent to use either the log posterior ratio or the measurement as the decision variable, and we choose the latter.

To make a decision, the observer responds “present” when the measurement exceeds the criterion, i.e. when


(5) $$ x_{\text{trial}} \gt k. $$

We will refer to *k* as the “absolute criterion”. We also introduce a “relative decision criterion” *c*, which is a linear transformation of the absolute criterion *k*:


(6) $$\begin{aligned} c & = \frac{k-\frac{\mu}{2}}{\sigma}. \end{aligned}$$

The absolute criterion has the same units as *s*, *x*, *µ*, and *σ*, whereas the relative criterion is dimensionless.

For a summary of different SDT-related terms and the definitions we use in this paper, see Table 1.

**Table 1.** Definitions used in this paper, with worked expressions for the main task discussed, namely distinguishing a signal at *µ* from 0 in the presence of Gaussian noise.

Concept	Definition (for fixed signal in Gaussian noise)
Discriminability (unitless)	$d^{\prime}=z(H) - z(F) = \frac{\mu}{\sigma}$
Relative criterion (unitless)	$c = -\frac{z(H) + z(F)}{2} = -z(F) - \frac{d^{\prime}}{2} = \frac{k}{\sigma} - \frac{d^{\prime}}{2} = \frac{k - \frac{\mu}{2}}{\sigma}$
Absolute criterion (same unit as *σ*)	$k = -\sigma z(F) = \mu - \sigma z(H) = \sigma\left(c + \frac{d^{\prime}}{2}\right)$
Log posterior ratio (unitless)	$d = \log \frac{p(C=1)}{p(C=0)} + \frac{\mu}{\sigma^2}\left( x - \frac{\mu}{2}\right)$
Bayesian criterion on the measurement (same unit as *σ*)	$k_{\text{Bayes}} = \frac{\mu}{2} - \frac{\sigma^2}{\mu} \log \frac{p(C=1)}{p(C=0)}$
Bayesian relative criterion (unitless)	$c_{\text{Bayes}} = - \frac{\sigma}{\mu} \log \frac{p(C=1)}{p(C=0)}$

The model observer’s “sensitivity” is a signal-to-noise ratio:


(7) $$\begin{aligned} d^{\prime}& = \frac{\mu}{\sigma}. \end{aligned}$$

A higher *µ* (a larger-magnitude difference between “present” and “absent”) or a lower *σ* (less measurement noise) will result in less overlap between the “present” and “absent” distributions and higher sensitivity. The same *d*^ʹ^ could arise from an infinite number of combinations of *µ* and *σ*.

### Summary of Rahnev et al.’s experiment

Rahnev *et al*.’s experiment that we primarily consider consists of a detection task where attention is manipulated with visual cues. The screen is divided into four quadrants. Each quadrant displays either a patch of visual noise or a noisy Gabor patch. Each diagonal pair contains the same stimulus. Subjects are cued to attend to either diagonal pair. They are then probed to respond about whether they saw or did not see a Gabor, at either the cued or uncued locations. Trials on which the cued locations are probed for response are “valid” trials (on which attentional allocation is presumably high) and trials on which the uncued locations are probed were “invalid trials” (on which attentional allocation is presumably low).

In both valid and invalid conditions, experimenters measured the subjects’ hit rate *H* and false-alarm rate *F*. The authors use z-scores for *H* and *F* to compute sensitivity *d*^ʹ^ and relative criterion *c* according to the standard formulae from signal detection theory:


(8) $$\begin{aligned} d^{\prime}& = z(H) - z(F), \end{aligned}$$

(9) $$\begin{aligned} c & = - \frac{z(H) + z(F)}{2}. \end{aligned}$$

The authors titrate the contrast of the stimuli such that in approximation


(10) $$\begin{aligned} d^{\prime}_{\text{val}} = d^{\prime}_{\text{inv}}, \end{aligned}$$

where “val” and “inv” refer to the subsets cue condition (valid or invalid). The empirical finding can then be summarized as


(11) $$ c_{\text{inv}} \lt c_{\text{val}}. $$

In words, the *relative* decision criterion in the invalid condition was measured to be smaller than in the valid condition. Based on this finding, what can we infer about the *absolute* criterion in either attentional condition? We show in the next section that this finding is insufficient to infer a fixed absolute criterion.

## Degeneracy of solutions

### Inequality Accounting for Rahnev’s Findings (with *d’* matched)

There are a number of combinations of absolute criteria and noise parameters that are consistent with the empirical findings in Experiment 1 of Rahnev *et al*. 2011. Each observer measurement is made under one of two conditions: valid (attended) and invalid (unattended). Therefore, we first allow *µ* and *σ* to be condition-dependent and correspondingly attach labels “val” and “inv”:


(12) $$\begin{aligned} d^{\prime}_{\text{val}} & = \frac{\mu_{\text{val}}}{\sigma_{\text{val}}}, \end{aligned}$$

(13) $$\begin{aligned} c_{\text{val}} & = \frac{k_{\text{val}}-\frac{\mu_{\text{val}}}{2}}{\sigma_{\text{val}}}, \end{aligned}$$

(14) $$\begin{aligned} d^{\prime}_{\text{inv}} & = \frac{\mu_{\text{inv}}}{\sigma_{\text{inv}}}, \end{aligned}$$

(15) $$\begin{aligned} c_{\text{inv}} & = \frac{k_{\text{inv}}-\frac{\mu_{\text{inv}}}{2}}{\sigma_{\text{inv}}}. \end{aligned}$$

We now assume that in the invalid condition, when attention is lower, the level of measurement noise (i.e. the trial-to-trial variance in the observer’s decision variable) is higher. In other words, $\sigma_{\text{inv}}\gt\sigma_{\text{val}}$. Eq. (10) can be reformulated:


(16) $$\begin{aligned} \frac{\mu_{\text{inv}}}{\sigma_{\text{inv}}} &= \frac{\mu_{\text{val}}}{\sigma_{\text{val}}}. \end{aligned}$$

Rahnev’s empirical finding, per Eq. (11), can be reformulated as:


(17) $$\begin{aligned} \frac{k_{\text{inv}}-\frac{\mu_{\text{inv}}}{2}}{\sigma_{\text{inv}}} &\lt \frac{k_{\text{val}}-\frac{\mu_{\text{val}}}{2}}{\sigma_{\text{val}}}, \end{aligned}$$

(18) $$\begin{aligned} \frac{k_{\text{inv}}}{\sigma_{\text{inv}}}-\frac{\mu_{\text{inv}}}{2\sigma_{\text{inv}}} &\lt \frac{k_{\text{val}}}{\sigma_{\text{val}}}-\frac{\mu_{\text{val}}}{2\sigma_{\text{val}}}. \end{aligned}$$

At this point, it is helpful to consider the range of *k*_val_. Empirically, Rahnev *et al*. find that $c_{\text{val}}\gt0$, which by Eq. 6 implies that $k_{\text{val}}\gt0$. (In fact, the necessary and sufficient condition for the latter is that $c_{\text{val}}\gt-\frac{d^{\prime}}{2}$, which is by extension also empirically true.) Henceforth, we will assume that $k_{\text{val}}\gt0$.

Combining Eqs. (16) and (17), we find


(19) $$ k_{\text{inv}} \lt \frac{\sigma_{\text{inv}}}{\sigma_{\text{val}}}k_{\text{val}}, $$

(20) $$ k_{\text{val}} \gt 0. $$

Thus, in the signal detection theory model of the task, any combination of *k*_inv_, $k_{\text{val}}\gt0$, $\sigma_{\text{inv}}$, and $\sigma_{\text{val}}$ that satisfies Eqs. (19) and (20) can explain the experimental findings for detection tasks. We will refer to Eq. (19) as the “Inequality Accounting for Rahnev’s Findings”, or the IARF for short. Throughout this paper, we will assume that Eq. (20) holds for stimulus detection tasks.

### Rahnev solution

The authors next propose a kind of explanatory model, which, following Gorea (Gorea and Sagi 2000), they call a “unified criterion” model. In this model, the observer uses the same, fixed measurement criterion in both conditions, i.e.


(21) $$ k_{\text{fixed, inv}} = k_{\text{fixed, val}}. $$

This is one solution to Eq. (19), since we would have


(22) $$ 0 \lt k_{\text{fixed, inv}}=k_{\text{fixed, val}} \lt \frac{\sigma_{\text{inv}}}{\sigma_{\text{val}}}k_{\text{val}}. $$

### All solutions

Rahnev’s fixed-criterion model is far from the only solution to Eq. (19). In particular, *k*_inv_ could be different from ${k_{\text{val}}}$, as long as it is smaller than $\frac{\sigma_{\text{inv}}}{\sigma_{\text{val}}}{k_{\text{val}}}$. This means that the experimental results are consistent with a wide range of models whose measurement criteria are in fact sensitive to uncertainty. For example, even in the scenario that $\sigma_{\text{inv}}\gt\sigma_{\text{val}}$, one could imagine an observer who scales their measurement criterion by the square root of the noise level. Then,


(23) $$ k_{\text{sqrt, inv}}= \sqrt{\frac{\sigma_{\text{inv}}}{\sigma_{\text{val}}}} k_{\text{sqrt, val}}\lt \frac{\sigma_{\text{inv}}}{\sigma_{\text{val}}} k_{\text{sqrt, val}}, $$

where we take advantage of the fact that the square root of a positive number greater than 1 is smaller than the number itself. Thus, the IARF is satisfied even despite the measurement criterion shifting.

**Figure 1.**
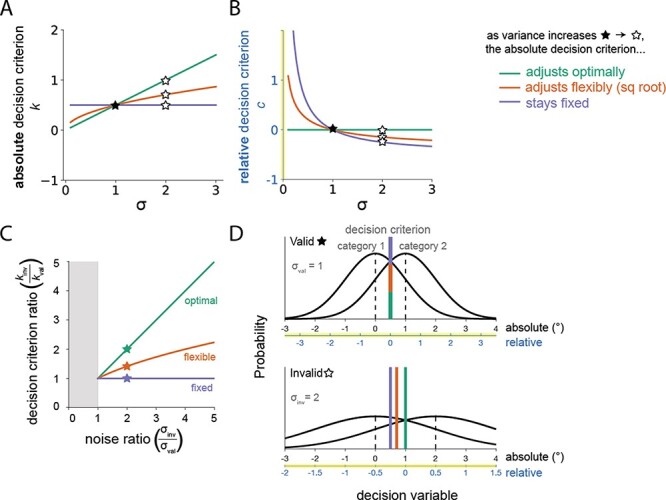
Three possible relationships between the trial-to-trial variance of the decision variable *σ* and an observer’s absolute (A) or relative (B) decision criterion. On a detection or coarse discrimination task, we assume that inattention leads to an increase in the trial-to-trial variability of the decision variable (i.e. an increase in *σ* from valid (filled star) to invalid (open star) conditions). Here, we assume an increase from $\sigma_{\text{val}}=1$ to $\sigma_{\text{inv}}=2$ (where the decision variable distributions across conditions are pictured in panel D). Adherents to the fixed criterion assumption believe that the absolute criterion remains fixed as *σ* increases (purple line, panel A). This corresponds to a decreasing relative criterion (i.e. a more “liberal” relative criterion) as variability increases (purple curve, panel B). An observer might alternatively scale their criterion by the square root of the variance (orange, panel A). This would similarly account for the empirical finding of a monotonically decreasing (i.e. more liberal) relative decision criterion in a high-*σ* compared to a low-*σ* condition (orange curve, panel B). Lastly, the Bayes-optimal solution (Appendix A) entails optimal sensitivity of the absolute decision variable *k* as *σ* increases (green line, panel A), which corresponds to a fixed relative criterion in a *σ*-normalized space (green line, panel B). (C) Visualizing the space of possible absolute criterion and decision variable variance ratios that satisfy the IARF. Any combination of parameters below the green optimal line would satisfy the IARF. The fixed criterion observer posited by Rahnev (purple) is given by the purple line with a fixed criterion ratio of 1. Any point above the purple line would violate the fixed criterion assumption. An observer who scales their measurement criterion by the square root of the noise level (orange) also satisfies the IARF and serves as just one of many possible examples of suboptimal, non-fixed models that do so. Anything between the opt line and fixed line suggests a shifting criterion in the correct direction which falls short of the optimal magnitude. The gray zone represents combinations where invalid noise is smaller than valid noise, which we assume is not possible. The zone below the fixed line, where invalid criterion is lower than the valid criterion, while unlikely, would still be consistent with the IARF. (D) Shifts in the decision criteria from valid (top) to invalid (bottom) conditions in an example case where $\sigma_{\text{val}}=1$ and $\sigma_{\text{inv}}=2$. Decision variable distributions are given by the black curves. The decision criteria used to separate category 1 from category 2 are given by vertical lines of their respective colors. The decision variable distributions and decision criteria can be expressed in a normalized, relative space (lower x-axis), in which the Bayes-optimal decision criterion is fixed at 0. They can also be expressed in an absolute decision variable space, which we assume to be equal to degrees in a coarse discrimination task. In absolute decision variable space, the fixed criterion model suggests a decision criterion (purple) which stays fixed from valid to invalid conditions (resulting in a relative decision criterion which is said to shift leftward). Here we show a fixed criterion observer which happens to be optimal in the valid condition, but those observers which set a single suboptimal criterion for both valid and invalid conditions is also fixed.

In panels A and B of Figure 1, we visualize three possible relationships between the trial-to-trial variance of the decision variable (*σ*) and an observer’s absolute (*k*, panel A) or relative (*c*, panel B) decision criterion. Inattention could lead to an increase in the trial-to-trial variability of the decision variable on a detection or discrimination task, i.e. an increase in *σ*. Fixed criterion models assume that the absolute criterion remains fixed as *σ* increases (purple line, panel A). This corresponds to a decreasing relative criterion as variability increases (purple curve, panel B)—in other words, with fixed criteria, we should expect the relative criterion to be more liberal in unattended (high *σ*) compared to the attended (low *σ*) conditions. There are, however, many other relationships that could equally satisfy this inequality—for example, an observer might scale their criterion by the square root of the variance (orange, panel A). This would similarly account for the empirical finding of a monotonically decreasing (i.e. more liberal) relative decision criterion in a high-*σ* compared to a low-*σ* condition (orange curve, panel B). Lastly, the Bayes-optimal solution entails optimal sensitivity of the absolute decision variable *k* as *σ* increases (green line, panel A), which corresponds to a fixed relative criterion in a *σ*-normalized space (green line, panel B).

From (19), we visualize the space of possible absolute criterion and decision variable variance ratios that satisfy the IARF in [Figure 1c]. Any combination of parameters below the green opt line would satisfy the IARF. The fixed criterion observer posited by Rahnev is given by the purple line, with a fixed criterion ratio of 1. Any point above the purple line would violate the unified criterion assumption. An observer who scales their measurement criterion by the square root of the noise level (orange line) also satisfies the IARF and serves as just one of many possible examples of suboptimal, non-fixed models that do so. Anything between the opt line and fixed line suggests a shift in the criterion in the correct direction, but one which falls short of the optimal magnitude.

We should moreover leave open the possibility that even a fully Bayesian observer who has wrong beliefs about certain features of the generative model can also satisfy the IARF. We demonstrate this possibility in Appendix B.

## Tasks that distinguish between a fixed and a flexible criterion

Since we are interested in distinguishing between a fixed and flexible criterion in observers, the goal is to determine and compare the values of *k*_val_ and *k*_inv_. But as we have demonstrated above, finding a unique solution for *k* is non-trivial on most task designs. We propose that two experimental requirements must be met when testing the fixed-criterion hypothesis: Firstly, the decision variable stimulus must be plausibly identifiable with some known stimulus-derived feature axis. Secondly, the task design must be such that subjects have an incentive to shift their criterion as uncertainty changes. We will now elaborate on these requirements.

### Requirement 1: A determinate mapping from the stimulus space to the decision variable space

We propose that the problem we ran into in the previous section is an example of a more general problem with standard signal detection theory paradigms, which we call the “indeterminate mapping” problem. The problem is that, in most signal detection theoretic tasks, we do not know how an observer maps the stimulus variable space that the experimenter can access and manipulate (orientation, contrast, and luminance) to the observer’s own internal decision variable space, in order to make decisions in the task at hand. The observer’s decision variable space may reflect some non-linear warping of the stimulus variable space of interest or may reflect a different stimulus variable space altogether than what the experimenter had in mind. For instance, an experimenter may manipulate units of contrast in a Gabor detection task, but the observer’s decision variable might instead reflect units of luminance for a given patch of pixels. Even more subtly, the observer’s decision variable may scale with contrast but in some non-linearly compressive way, for example, obeying a relationship characterized by the Weber–Fechner law, whereby a unit increase in a stimulus property like contrast or luminance may result in a non-linear increase in an observer’s decision variable, depending on the absolute magnitude of the property. Such examples illustrate the need to carefully rule out plausible alternatives before making conclusions about what physical properties of the stimulus subjects use as the basis for their decision variable. We cannot pin down the trial-to-trial mean of an observer’s decision variable (*µ*) unless we are able to justify our assumption about the observer’s mapping from stimulus feature to decision variable.

We run into the problem of a degeneracy of criterion solutions for *k* as a result of our inability to pin down a definite value for *µ*.

Recall that Eq. (6)


$$\begin{aligned} c & = \frac{k-\frac{\mu}{2}}{\sigma} \end{aligned}$$

shows that *c* is the distance between the measurement criterion and the optimal criterion, also expressed in units of standard deviation. Likewise, Eq. (7)


$$\begin{aligned} d^{\prime}& = \frac{\mu}{\sigma} \end{aligned}$$

shows that *d*^ʹ^ is the distance between the means of the signal and noise distributions, in units of standard deviation. The two relative quantities *d*^ʹ^ and *c*, which we can measure in an experiment, are therefore expressed in terms of three absolute quantities *µ*, *σ*, and *k*, which we would like to infer. We cannot infer three variables from two measurements, so if we do not know (or cannot plausibly assume to know) either *µ* or *σ*, *k* will remain fundamentally unidentifiable, leading to the indeterminacy problem described in the above section. (See Denison et al. 2018, Appendix 1).

Thus, in order to pin down the decision criterion *k*, we must first pin down the mean of the decision variable *µ*. And in order to pin down *µ*, we need to design a task that allows us to plausibly assume that *µ* is identical to some stimulus feature. This requirement is not met by the standard stimulus detection task (as used in Rahnev 2011) because we do not know the mapping between the experimenter-set contrast and the observer’s internal decision variable for detection. Importantly, we have no way of knowing how attention changes the decision variable. Therefore, in detection tasks, we cannot say how changes in either physical stimulus contrast or attention lead, over many trials, to changes in *µ* or *σ* in decision variable space.

Simple orientation discrimination tasks (i.e., coarse discrimination between -45^°^ and 45^°^ or fine discrimination between -2^°^ and 2^°^) also fall victim to the mapping problem because there are alternative decision variable axes that subjects may plausibly use other than orientation. If the subject’s decision variable were faithful to orientation, each unit on the decision variable axis would correspond to a different orientation in degrees (where the half-way point between -45^°^ and 45^°^ is an orientation of 0^°^). This could be implemented by reading the difference in firing rates between two neural populations, each of which is maximally responsive to the stimulus orientation corresponding to the one of the two stimulus values. In general, however, we are unable to rule out any such scheme that involves a decision variable, which is low at one extreme (corresponding to stimulus 1), high at the other extreme (corresponding to stimulus 2), and monotonically increasing. And any such axis could be implemented by the difference in firing rates between two neural populations ($\Delta r$), where each population fires maximally for their preferred stimulus feature. For example, an alternative possibility to orientation is that the subject’s decision variable reflects a graded linear interpolation in pixel space between -45^°^ and 45^°^ (where the half-way point in the decision variable space reflects an equal superimposition of an image of -45^°^ and 45^°^), with two populations maximally sensitive to either extreme. In simple discrimination tasks, we are unable to rule out the former scenario in favor of the latter nor are we able to rule out nonlinear variations of either scenario that preserve a monotonic mapping.

We suggest that the most promising approach for encouraging observers to use a particular axis is to present a “continuum” of stimuli along the desired decision variable axis and to ask subjects to categorize the stimuli into one of two learned category “distributions”. For example, subjects could categorize an oriented Gabor as drawn from one of two overlapping normal distributions with different means (e.g. ±5^°^) and the same standard deviation (e.g. 8^°^), as was done in (Rahnev 2021). In requiring subjects to distinguish between distributions of orientations (and not merely two fixed orientations, as in traditional discrimination tasks), these tasks provide stronger justification for the assumption that the decision variable is in fact identical to orientation, since orientation is the simplest plausible variable that accurately reflects strength of evidence for category delineation.

Zak et al. (2012) and Rahnev (2021) both addressed the issue of decision variable identifiability using external noise. These studies, along with Kontsevich et al. (2002), recognized that the experimenter’s lack of knowledge about the observer’s internal noise distribution limited the interpretability of the findings of Gorea and colleagues (Gorea and Sagi 2000, 2001) that first led to the notion of a unified criterion. They provide examples of how experimenters can identify an observer’s absolute decision criterion using a task in which external noise is significantly greater than internal noise, making internal noise a negligible component of the observer’s internal response variance. Both studies generally observed flexible but suboptimal criterion adjustment, which they referred to as “criteria attraction”, rather than a single, fixed criterion, though with some variability across observers in Zak *et al*. Observers who were informed about the experimental design had behavior that was closer to optimal than those who were not informed (Zak et al. 2012), indicating the importance of ensuring that an observer’s knowledge matches experimenter assumptions when evaluating the optimality of behavior (Rahnev and Denison 2018).

In sum, we propose that we can only pin down *µ* if we can assert a one-to-one mapping between a known physical stimulus space and an unobservable decision variable space. It is important to seek a task design where subjects are as limited as possible to using only the decision variable mapping intended by the experimenter. One way to achieve this is by using an external noise approach: presenting a continuum of stimuli along the desired decision variable axis and asking subjects to identify stimuli as belonging to different category distributions along this axis.

### Requirement 2: Built-in incentive to shift the decision criterion based on uncertainty

Secondly, even if we had full knowledge of the mapping between a stimulus feature and the subject’s decision variable, an additional condition for resolving the effect of uncertainty on the decision (Qamar et al. 2013, Ma et al. 2011, Keshvari et al. 2012, Trommershauser et al. 2011, Ma 2012, Zhou et al. 2020, Yoo et al. 2021). For instance, in any discrimination or categorization task in which the category distributions are symmetric and offset (e.g. the internal measurement distributions for the two categories have the same variance and means +/- *µ*), the optimal decision variable is at the intersection point of the two internal measurement distributions. This point stays the same as uncertainty changes (Fig. 2) panel A, so observers have no incentive to shift their decision criterion. In this situation, we would not expect differences in *k*_inv_ and *k*_val_ from an optimal observer. (See Denison et al. (2018), Appendix 1.) In trying to falsify the notion that observers do not shift their criterion, it is important to provide through task design the incentive to shift. Thus, to study whether uncertainty affects decision criteria, a task should provide an optimal observer with an incentive to shift their decision criterion when uncertainty changes.

### The embedded category task

The “embedded category” task (Fig. 2, panel B; (Qamar et al. 2013); (Denison et al. 2018); (Adler and Ma 2018)) meets both criteria for distinguishing between a fixed and flexible criterion. To infer attention-dependent shifts in *k* based on measurements of *d*^ʹ^ and *c*, we have outlined that it is necessary to know either *µ* or *σ* and to provide a task structure that incentivizes observers to respond to increasing uncertainty by shifting *k*—the embedded category task succeeds on both counts.

In the embedded category task, observers categorize a stimulus drawn from one of two distributions (categories) with the same mean but different standard deviations (Fig. 2, panel B). For example, in an orientation categorization version of this task, observers would categorize a given orientation as drawn from either a narrow distribution (category 1) or a broad distribution (category 2), both centered around horizontal (0 degrees). When the orientation of the test stimulus is near horizontal, it is more likely to belong to category 1, whereas when the orientation is far from horizontal, it is more likely to belong to category 2.

This task structure avoids the indeterminate mapping problem because orientation is the only plausible decision variable—it varies continuously from trial to trial and serves as the sole basis for category delineation. Since *µ* is known in physical units, and since *µ* is not expected to change when we manipulate the uncertainty of the stimulus by changing its physical contrast (Qamar et al. 2013, Adler and Ma 2018) or level of attention (Denison et al. 2018), it is therefore possible for us to infer the absolute criterion *k* from task performance.

Moreover, the embedded category task, by virtue of having unequal category stimulus variance, incentivizes subjects to shift their decision boundary as measurement uncertainty increases (see Fig. 2, panel B). The optimal decision boundary should shift with changes in uncertainty, satisfying our second requirement (Fig. 2, panel B, green line). Therefore, by plausibly fixing *µ* and *σ* and by incentivizing an uncertainty-dependent decision boundary, Denison *et al*. satisfy both minimum requirements for resolving the question of whether the absolute criterion, *k*, is fixed (Fig. 2, panel B, purple line) or flexible (Fig. 2, panel B, green line).

To briefly summarize Denison *et al*.’s results, they find that *k* was not fixed but instead adjusted flexibly based on level of uncertainty, in line with previous findings that the decision criterion is broadly sensitive to uncertainty (Qamar et al. 2013, Denison et al. 2018, Adler and Ma 2018) [see Denison *et al*., 2018, figure 4]. However, the degree to which the criterion shift is suboptimal is still very much an open question, and studies like Rahnev (2021) have begun to describe the phenomenon in more detail.

Similar results have been found in peripheral viewing tasks. For instance, Zhou et al. (2020) asked subjects to make decisions about the co-linearity of horizontal lines at varying peripheral eccentricities (i.e. at varying degrees of sensory uncertainty). In line with much of the probabilistic perception literature, the study suggests that subjects perform nearly optimally in setting decision criteria to correspond to each level of visual eccentricity (i.e. each level of uncertainty), suggesting that uncertainty-dependent criterion-setting may also be involved in subjective inflation of visual perception at the periphery. This poses a challenge to Solovey *et al*., 2015, in which the authors only put forward a fixed criterion model to explain the phenomenon (Solovey et al. 2015)).

### Other potential designs

The embedded category task is far from the only experimental design that can meet the two task requirements to test whether decision criteria are sensitive to attention-dependent uncertainty (or other forms of uncertainty more broadly). The question can be answered by modifying existing experimental designs. For example, an auditory-visual cue combination task in which subjects are asked to report the location of an object, and in which uncertainty is manipulated via valid or invalid cuing, should help shed further light on the question of flexible criteria. This is because, unlike a standard 2AFC detection or coarse discrimination task, the uncertainty manipulation should map to the observer’s decision variable space in a fairly predictable way (i.e. we should not expect inattention to systematically warp the observer’s internal decision space if that decision variable reflects spatial location) and because unequal measurement variance between the sensory modalities is expected to result in a shift in criterion as uncertainty is manipulated. Here, the unequal measurement variance caused by differences in reliability between visual and auditory information would play an analogous role to the unequal category variances in the embedded category task. Cue combination tasks typically involve estimates over a continuous variable (e.g. location) but can be converted into a decision-making task through comparison of that estimate to a standard cue (e.g. is the stimulus to the left, right, or straight ahead, relative to a standard cue?). There is a paucity of research in the cue combination domain that manipulates uncertainty through attention rather than a stimulus-driven manipulation (e.g. manipulations cue reliability). Cue combination tasks with an attentional manipulation paired with neural recording can also help shed light on the neural underpinnings of criterion-setting (for instance, a variant of Gu et al. (2008)). Additionally, some of the studies in which people use trial-to-trial sensory uncertainty information without trial-to-trial feedback may be suitable for modification to probe the effects of attention-dependent uncertainty on observer estimates and decision criteria.

Introducing unequal rewards between the two categories could also introduce an incentive to shift the decision criterion under increased uncertainty. The basic design could be similar to Whiteley and Sahani (2008), in which observers did a left–right categorization task under unequal rewards for left and right. Rather than manipulating reward, one could fix the rewards for either choice (while keeping them unequal) and manipulate attention. If attention acts only on the sensory noise level *σ*, then the reward-maximizing criterion will be attention-dependent. The same effect would be obtained by assigning different base rates (prior probabilities) to the two categories (Morales et al. 2015). However, these manipulations introduce an extra ingredient and further assumptions compared to the embedded category task. In these tasks, observers need to both learn the prior or reward information and combine that information with the likelihood. Only then would they have the potential to appropriately adjust their decision criterion as a function of attention-dependent uncertainty.

## Conclusion

In this paper, we have shown that the fixed-criterion model of subjective inflation requires re-thinking in light of evidence that decision criteria flexibly adjust according to uncertainty. Both fixed and flexible criteria are consistent with Rahnev *et al*.’s empirical findings, and indeed an infinite set of relations between the observer’s trial-to-trial decision variable and decision criterion could account for these results. Moreover, we have shown that not all task designs are suitable for demonstrating flexible criterion-setting because multiple plausible decision variable mappings may exist for a given task. Using an embedded category task design which allows for the presentation of intermediate stimuli along the desired feature axis encourages observers to use orientation rather than any other stimulus feature as the basis for their decision variable. A distinct advantage of distribution-based categorization tasks is that we can make better assumptions about the *µ* and *σ* of the observer’s internal decision variable distributions to make better inferences about the absolute decision criterion *k*. Moreover, if we are interested in investigating the sensitivity of an observer’s decision criterion to attention-dependent uncertainty, we should prefer experimental designs where there is an accuracy incentive for observers to shift their criterion as the trial-to-trial variance of their decision variable changes. Denison *et al*.’s embedded category task met these requirements and yielded evidence for attention-dependent uncertainty. This finding should be incorporated into future models of subjective inflation. An important future experimental direction is to develop a perceptual “detection” task, more aligned with those used to investigate subjective inflation, that meets the requirements described in this paper and assess anew whether perceptual criteria are fixed or flexible.

## Code

MATLAB code for reproducing the figures in this paper is available at https://github.com/WeiJiMaLab/fixed-flexible-criterion.

## Appendix A Optimal Bayesian observer

Under what condition is the IARF satisfied by a Bayesian observer? Bayesian models are a subset of signal detection theoretic models in which the criterion is not any arbitrary number, but is instead set to maximize expected accuracy (or another form of expected utility). A Bayesian observer uses particular pre-existing beliefs about the world to make inferences. If these beliefs are correct—i.e. reflect the true generative model of the observations—then expected accuracy will in fact be maximized. In the present section, we will treat that case; in the following section, we will allow for wrong beliefs.

Let us say that on a given trial, the measurement of the stimulus is *x*_trial_. The Bayesian observer uses knowledge of the generative model defined by Equations (1) and (2) to make the best possible judgment about the stimulus *s* given *x*_trial_. The “best possible” judgment here means the one that will maximize accuracy over many trials. This requires choosing the category (“present”, $s=\mu$ or “absent,” *s* = 0) with the highest posterior probability. To formalize this, we define the Bayesian observer’s internal decision variable *d* as the *log posterior ratio*

(A. 1)
$$ d \equiv \log \frac{p(s=\mu|x_{\text{trial}})}{p(s=0|x_{\text{trial}})}. $$

This can be written as the sum of the log likelihood ratio and the log prior ratio:

(A. 2)
$$ d = \log \frac{p(x_{\text{trial}}|s=\mu)}{p(x_{\text{trial}}|s=0)} + \log \frac{p(s=\mu)}{p(s=0)}. $$

Substituting Eqs. (1) and (2), we find

(A. 3)
$$ d = \frac{{\mu}\left(x_{\text{trial}}-\frac{\mu}{2}\right)}{{\sigma}^2}. $$

Reporting the stimulus with the highest posterior probability means reporting “present” when *d* > 0, or in other words, when $x_{\text{trial}}\gt\frac{\mu}{2} $. Comparing this with Eq. (5), we find that the optimal Bayesian criterion is

(A. 4)
$$ k_{\text{opt}} = \frac{\mu}{2}. $$

We now apply this equation to the comparison between valid and invalid conditions:

(A. 5)
$$ k_{\text{opt, inv}} = \frac{\mu_{\text{opt, inv}}}{\mu_{\text{opt, val}}}k_{\text{opt, val}}. $$

Since experimenters ensure that *d*^ʹ^ is matched between the valid and invalid conditions, this is equivalent to

(A. 6)
$$ k_{\text{opt, inv}} = \frac{\sigma_{\text{opt, inv}}}{\sigma_{\text{opt, val}}}k_{\text{opt, val}}. $$

This does *not* satisfy the IARF, Eq. (19), since that is a strict inequality. Stated plainly, Rahnev’s empirical findings suggest that people are not Bayes-optimal in the task, which the authors rightly emphasize. As measurement noise increases, observers do not adjust their decision criterion by the Bayes-optimal amount—adjustments fall short of this magnitude.

Additionally, we can easily rule out another general class of suboptimal models: If $k=\alpha \mu$ or $k = \alpha \sigma$, where *α* is an arbitrary constant, then the IARF is also not satisfied.

## Appendix B Bayesian observer with wrong beliefs

So far, we have considered an optimal Bayesian observer who holds correct beliefs about the generative model. While such an observer is not consistent with the data, a Bayesian observer who holds incorrect beliefs about the generative model might still be. Such an observer is said to have “model mismatch”—their internal model of the world is mismatched in some way to the task’s true generative model. A Bayesian observer with model mismatch will of course be, strictly speaking, “suboptimal”. Suboptimalities in inference have been proposed to account for variability in human behavior in other contexts (Beck et al. 2012, Drugowitsch et al. 2016, Shen and Ma 2016). Specifically, we allow here that an observer may have wrong beliefs about *µ*, *σ*, and *π*, the prior probability that the stimulus is present. We denote the observer’s belief about the stimulus value when the stimulus is present by $\tilde{\mu}$, their belief about noise level by $\tilde{\sigma}$, and their belief about the probability that the stimulus is present by $\tilde\pi$. Then, Eq. (A. 2) for the log posterior ratio (*d*) becomes

(B. 1)
$$\begin{aligned} d & = \frac{\tilde{\mu}\left(x_{\text{trial}}-\frac{\tilde\mu}{2}\right)}{\tilde{\sigma}^2} + \text{LPR}, \end{aligned}$$

where we define the “believed log prior ratio” LPR as

(B. 2)
$$ \text{LPR} \equiv \log \frac{\tilde{\pi}}{1-\tilde{\pi}}. $$

The decision rule of responding “present” when *d* > 0 now becomes

(B. 3)
$$ x_{\text{trial}} \gt \frac{\tilde \mu}{2} + \frac{\tilde\sigma^2}{\tilde\mu} \text{LPR}. $$

Comparing with Eq. (5), we see that the Bayesian criterion is

(B. 4)
$$ k= \frac{\tilde \mu}{2} + \frac{\tilde\sigma^2}{\tilde\mu} \text{LPR}. $$

Eq. (B. 4) indicates that for the Bayesian observer, the criterion is not free but is fully determined by the parameters that describe the observer’s beliefs over the structure of the world. The invalid and valid Bayesian criteria are

(B. 5)
$$\begin{aligned} k_{\text{Bayes, inv}} & = \frac{\tilde \mu_{\text{inv}}}{2} + \frac{\tilde\sigma_{\text{inv}}^2}{\tilde\mu_{\text{inv}}} \text{LPR}, \end{aligned}$$

(B. 6)
$$\begin{aligned} k_{\text{Bayes, val}} & = \frac{\tilde \mu_{\text{val}}}{2} + \frac{\tilde\sigma_{\text{val}}^2}{\tilde\mu_{\text{val}}} \text{LPR}. \end{aligned}$$

These expressions can be rewritten as

(B. 7)
$$\begin{aligned} k_{\text{Bayes, inv}} & = \tilde\sigma_{\text{inv}} \left( \frac{\tilde{d^{\prime}}_{\text{inv}}}{2} - \frac{\text{LPR}}{\tilde{d^{\prime}}_{\text{inv}}} \right), \end{aligned}$$

(B. 8)
$$\begin{aligned} k_{\text{Bayes, val}} & = \tilde\sigma_{\text{val}} \left( \frac{\tilde{d^{\prime}}_{\text{val}}}{2} - \frac{\text{LPR}}{\tilde{d^{\prime}}_{\text{val}}} \right), \end{aligned}$$

where we defined the *believed* sensitivities

(B. 9)
$$\begin{aligned} \tilde{d}^{^{\prime}}_{\text{val}} & \equiv \frac{\tilde{\mu}_{\text{val}} }{\tilde{\sigma}_{\text{val}}}, \end{aligned}$$

(B. 10)
$$\begin{aligned} \tilde{d}^{^{\prime}}_{\text{inv}} & \equiv \frac{\tilde{\mu}_{\text{inv}} }{\tilde{\sigma}_{\text{inv}}}. \end{aligned}$$

We now examine the circumstances under which a Bayesian observer with wrong beliefs sets criteria that satisfy the IARF, Eq. (19). For such an observer, the IARF can be rewritten as

(B. 11)
$$\begin{aligned} \frac{\tilde\sigma_{\text{inv}}}{\sigma_{\text{inv}}} \left( \frac{\tilde{d^{\prime}}_{\text{inv}}}{2} - \frac{\text{LPR}}{\tilde{d^{\prime}}_{\text{inv}}} \right) & \lt \frac{\tilde\sigma_{\text{val}}}{\sigma_{\text{val}}} \left( \frac{\tilde{d^{\prime}}_{\text{val}}}{2} - \frac{\text{LPR}}{\tilde{d^{\prime}}_{\text{val}}} \right). \end{aligned}$$

There are many combinations of $\tilde\pi$, $ \tilde{d}^{^{\prime}}_{\text{inv}}$, $ \tilde{d}^{^{\prime}}_{\text{val}}$, $ \tilde\sigma^{\prime}_{\text{inv}}$, and $ \tilde\sigma^{\prime}_{\text{val}}$ for which Eq. (B. 11) holds. A few special cases are of interest.

### Special Case 1

Firstly, if the observer uses the correct *σ*s and the correct value of the prior, $\tilde\pi = 0.5$, then $\text{LPR} = 0$ and Eq. (B. 11) becomes

(B. 12)
$$ \tilde{d}^{^{\prime}}_{\text{val}}\gt \tilde{d}^{^{\prime}}_{\text{inv}}. $$

Thus, wrong beliefs about sensitivity could be solely responsible for Rahnev’s findings. Even though experimenters matched *d*^ʹ^ across conditions, it may be the case that subjects incorrectly believe their sensitivity to be higher for the valid compared to the invalid conditions.

### Special Case 2

Secondly, if the observer correctly believes that $\tilde{d}^{^{\prime}}_{\text{val}} = \tilde{d}^{^{\prime}}_{\text{inv}}$, but holds incorrect beliefs about either or both *σ*s, then Eq. (B. 11) reduces to

(B. 13)
$$\begin{aligned} \frac{\tilde\sigma_{\text{inv}}}{\sigma_{\text{inv}}} & \lt \frac{\tilde\sigma_{\text{val}}}{\sigma_{\text{val}}}. \end{aligned}$$

This means that the noise level in the valid condition (the lower noise level) is overestimated by a greater factor relative to its true value than the noise level in the invalid condition. (Or, equivalently, the noise level in the invalid condition is underestimated by a greater factor than the noise level in the valid condition.) Thus, wrong beliefs about internal noise in either or both conditions could also be solely responsible for Rahnev’s findings.

### Special Case 3

Thirdly, if the observer uses the correct *σ*s but $\tilde\pi$ is not necessarily equal to 0.5, and they incorrectly believe that $ \tilde{d}^{^{\prime}}_{\text{val}} \gt \tilde{d}^{^{\prime}}_{\text{inv}} $, then Eq. (B. 11) becomes

(B. 14)
$$\begin{aligned} \text{LPR} & \lt \frac{\tilde{d^{\prime}}_{\text{val}}\tilde{d^{\prime}}_{\text{inv}}}{2}. \end{aligned}$$

Since *d*^ʹ^s are positive, and since $\tilde\pi \leq 0.5$ results in a LPR $\leq 0$, this condition is always satisfied when $\tilde\pi \leq 0.5$. However, it also holds for higher values of $\tilde\pi$, up to a limit determined by the believed *d*^ʹ^s. An analogous scenario with opposite signs arises when the observer uses the correct *σ*s but incorrectly believes that $ \tilde{d}^{^{\prime}}_{\text{val}} \lt \tilde{d}^{^{\prime}}_{\text{inv}} $.

We conclude, firstly, that within the Bayesian framework, there are multiple sets of wrong beliefs that can account for Rahnev’s findings without positing a fixed decision criterion. Secondly, even within the Bayesian framework, Rahnev’s findings do not imply that the criterion is the same between the invalid and valid conditions.
